# Posterior parietal cortical areas and recovery after motor stroke: a scoping review

**DOI:** 10.1093/braincomms/fcad250

**Published:** 2023-09-28

**Authors:** Antonia Reibelt, Fanny Quandt, Robert Schulz

**Affiliations:** 1 Experimental Electrophysiology and Neuroimaging Lab, Department of Neurology, University Medical Center Hamburg Eppendorf, 20246 Hamburg, Germany; 1 Experimental Electrophysiology and Neuroimaging Lab, Department of Neurology, University Medical Center Hamburg Eppendorf, 20246 Hamburg, Germany; 1 Experimental Electrophysiology and Neuroimaging Lab, Department of Neurology, University Medical Center Hamburg Eppendorf, 20246 Hamburg, Germany

**Keywords:** connectivity, PPC, plasticity, stimulation, coupling

## Abstract

Brain imaging and electrophysiology have significantly enhanced our current understanding of stroke-related changes in brain structure and function and their implications for recovery processes. In the motor domain, most studies have focused on key motor areas of the frontal lobe including the primary and secondary motor cortices. Time- and recovery-dependent alterations in regional anatomy, brain activity and inter-regional connectivity have been related to recovery. In contrast, the involvement of posterior parietal cortical areas in stroke recovery is poorly understood although these regions are similarly important for important aspects of motor functioning in the healthy brain. Just in recent years, the field has increasingly started to explore to what extent posterior parietal cortical areas might undergo equivalent changes in task-related activation, regional brain structure and inter-regional functional and structural connectivity after stroke. The aim of this scoping review is to give an update on available data covering these aspects and thereby providing novel insights into parieto-frontal interactions for systems neuroscience stroke recovery research in the upper limb motor domain.

## Teaser

Reibelt *et al*. give an update on posterior parietal cortical areas and their changes in brain activation, regional brain structure and inter-regional structural, functional and effective connectivity and show how these changes relate to recovery processes after motor stroke.

## Graphical Abstract

**Graphical abstract:**
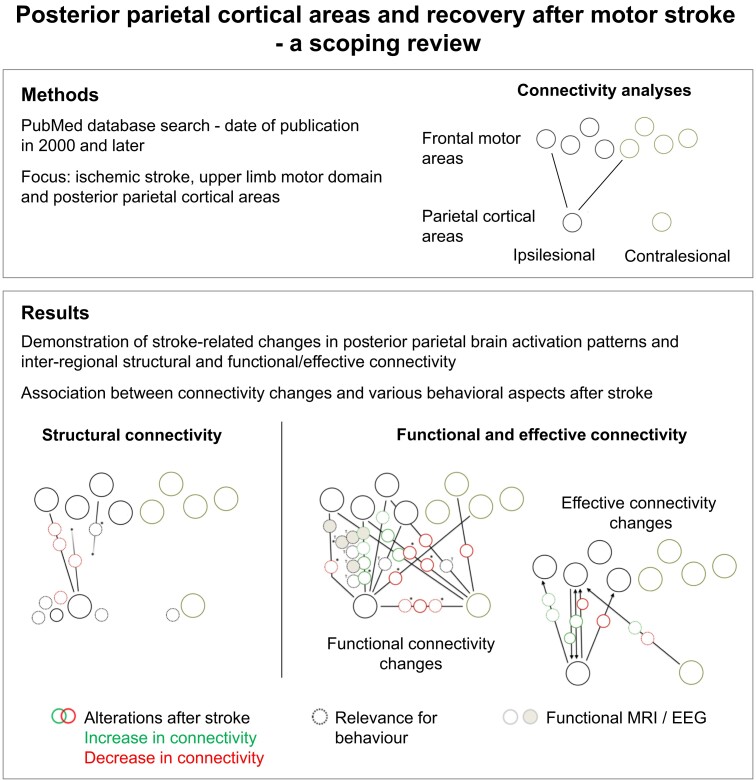

## Introduction and objective

Ischaemic stroke is one of the leading causes for persistent, long-term disability in western, industrialized countries.^1^ The number of stroke survivors in the European Union is expected to increase by 27% from 2017 to 2047^2^ with considerable societal direct and indirect costs of up to €60 billion across 32 European countries.^3^ During the last decades, brain imaging and electrophysiology have aimed at exploring mechanisms of recovery after stroke and provided valuable insights on how the brain reacts to acute focal lesions. Studies in the motor domain have illustrated how multiple brain areas of both hemispheres undergo time-dependent changes not only in brain structure and regional activation but also in multi-site inter-regional structural and functional connectivity and related these changes to recovery processes.^4-9^ Most evidence for recovery-related dynamics in regional and network brain structure and function is available for key motor areas of the frontal lobe, comprising the primary motor cortices (M1), the dorsal and ventral premotor cortices (PMC), and the supplementary motor areas (SMA). For instance, it has been shown that a stroke leads to early upregulation of ipsilesional and contralesional motor cortices with an extended recruitment of brain regions particularly in more impaired patients. Resting-state and task-related functional MRI studies have found alterations in the connectivity and information flow between these key motor areas and have linked them to recovery.^4^

In comparison to key motor areas of the frontal lobe, the involvement of posterior parietal cortical areas and their contribution to stroke recovery has not been in the focus for many years although these regions are similarly important for motor function in the healthy human brain. Recently, the field has increasingly started to explore to what extent posterior parietal cortical areas including areas along the intraparietal sulcus (IPS) on the superior (SPL) and inferior parietal lobules (IPL) might undergo equivalent changes after stroke in regional brain structure, task-related activation and inter-regional functional and structural connectivity. Studies in healthy participants have already evidenced that skilled voluntary movements of the upper limb, including reaching, gasping, object manipulation and tool use,^10^ significantly involve interactions between posterior parietal cortical areas and frontal motor areas. For instance, the anterior portion of the lateral bank of the IPS has been found to contribute to grasping,^11,12^ precision grip^13^ and grip force adaption.^14^ It provides PMC with visual information about object properties and influences PMC-M1 interactions to generate task-specific motor commands. Also, more posterior cortices along the IPS have been reported to show grasp-related activations^15^ and encode spatial object features relevant for movement planning and execution.^16,17^ Another study has related the success in motor skill acquisition to the state of parieto-frontal functional networks.^18^ One important structural substrate of these interactions is dense connections between M1, ventral PMC and anterior and caudal IPS via the superior longitudinal fascicles. These pathways have been traced in animals^19,20^ and humans.^17,21-23^

Collectively, as posterior parietal regions contribute to dexterity and skilled hand and arm movements in healthy participants and as they are connected to key motor areas of the frontal lobe, it has been hypothesized that these parietal brain areas might be also involved in recovery processes after stroke. The aim of this article was to review the available literature on stroke-related changes in regional and inter-regional brain function, structure and connectivity of posterior parietal cortical areas and their association with motor recovery after stroke.

### Literature search

For this scoping review article, PubMed database was scanned for primary and secondary literature including systematic reviews and meta-analyses with the date of publication in 2000 and later. Literature selection was biased towards studies on ischaemic stroke patients, the upper limb motor domain and the posterior parietal cortex. Hence, studies on aphasia or neglect and studies involving global network/connectome analyses were not in the scope of the present work. Study selection was finalized based on data quality, methods, and novelty. Results are organized and presented with a focus on three distinct aspects, (i) changes in regional brain activation and regional transcranial magnetic stimulation (TMS) inference of causal involvement, (ii) structural connectivity analyses, and (iii) functional and effective connectivity analyses.

## Data availability

Data sharing is not applicable to this article as no new data were created or analysed in this study.

## Results

### Regional brain activation and TMS perturbation studies of posterior parietal cortical areas

Most evidence for time- and recovery-dependent alterations in focal brain activation has been accumulated for key motor areas of the frontal lobe, comprising M1, dorsal and ventral PMC and SMA, as reported by systematic meta-analyses.^24,25^ Overactivation early after stroke, observed both in the ipsilesional and contralesional hemispheres and particularly in more severely impaired patients, normalizes over time. Normalizing activation patterns have been associated to motor recovery.

Compared to frontal motor areas, meta-analyses have reported only limited evidence of stroke-related dynamics in posterior parietal brain activation. As large inter-subject variability in stroke cohorts, e.g. regarding the level of impairment, lesion locations and motor tasks, might have biased the findings of activation likelihood estimations towards the frontal lobe,^24,25^ an in-depth analysis on the single study level seems to be warranted to draw a more finely grained picture of changes in brain activation in posterior parietal cortical areas after stroke. In fact, taking all stroke patients and time points into account, one meta-analysis found an increased brain activation in bilateral precentral gyrus, left postcentral gyrus and right superior frontal gyrus, cingulate gyrus and the paracentral lobule. Interestingly, when biasing the analysis towards patients with subcortical lesions and intact cortices, upregulated active regions additionally comprised the right supramarginal gyrus and IPL. Moreover, when contrasting the activation in the acute stage after stroke with data from the chronic stage, not only the precentral and postcentral gyrus showed an overactivation in the acute stage but also the left IPL.^25^ Such meta-analyses are based on multimodal approaches whose details are worth noting. For instance, positron emission tomography data have shown an overactivation particularly early after subcortical stroke in bilateral ipsilesional and contralesional SPL and IPL.^26-28^ Functional MRI has similarly reported a task-related bilateral overactivation in posterior parietal cortical areas,^29-34^ and some studies have evidenced correlations between regional activations and subsequent amount of recovery. For example, activation in the contralesional IPL and ipsilesional IPS negatively related to recovery.^35^ One recent study has emphasized that posterior parietal brain activation might depend on the level of impairment by showing an overactivation specifically in the contralesional parietal lobe in hemiplegic chronic stroke patients with a positive association with motor functions.^36^ Most studies have found an early overactivation with subsequent normalization over time. However, there are also contradictory data. For example, one longitudinal study in patients with isolated subcortical lesions reported an increasing activation in the ipsilesional inferior parietal cortex as time proceeded.^37^ In summary, most task-related brain activation studies have argued that posterior parietal cortical areas might activate more strongly early after stroke and most likely in patients with more severe deficits and, speculatively, the highest need of secondary motor areas to compensate for motor deficits. Compared to frontal motor areas, posterior parietal involvement appears to be much more variable and inconsistent across studies and different patient cohorts. Differences in the level of impairment, lesion locations, and selection of regions within the complex functional anatomy of the posterior parietal cortex might be influential factors.^38^ Also, inter-study variability might lie in the kind of motor tasks used which may drastically differ regarding their recruitment of parietal resources. Hence, this might result in highly variable findings, especially since most studies rely on relatively simple motor tasks rather than complex tasks involving reach and grasp components or fine motor skills of dexterity. In meta-analyses, this might explain the absence of consistent posterior parietal brain activation across studies, which, in turn, might also explain the absence of associations with behaviour.

Brain activation studies have the important limitation that their results cannot be interpreted as causal. In other words, longitudinal changes in brain activation can be driven by recovery rather than they are the cause of recovery. The same holds true for recovered hand function when addressed cross-sectionally. Hence, activity changes in posterior parietal areas might be simply an epiphenomenon without any functional importance for recovery or recovered hand function after stroke. Regarding this critical issue, few TMS studies have aimed at probing the influence of posterior parietal regions on recovered hand function by the perturbation of ongoing local activity. Contralesional SPL stimulation led to functional deficits of timing and accuracy in seven patients three months after isolated subcortical stroke.^39^ In contrast, in the acute phase of stroke, stimulation of the contralesional anterior IPS led to the opposite effect with an improvement in motor functions in 14 patients with cortical stroke.^33,40^ Such data further indicate that the contribution of posterior parietal cortical areas might be time-sensitive and specific to lesion locations and regions tested. To what extent beneficial effects under online perturbation can really be interpreted as a positive effect of stopping an ongoing maladaptive process is still under debate.^41^ Regarding prospective aspects of recovery over time, there are no TMS studies which, for instance, have probed the effect of repetitive perturbation or up- or downregulation of posterior parietal cortical areas early after stroke and followed the clinical course of recovery thereafter. Only such approaches will be able to answer whether posterior parietal areas might causally drive recovery or whether they might support recovered motor functions without driving recovery itself.

### Structural imaging and connectivity analyses of posterior parietal cortical areas

Structural imaging has significantly contributed to stroke recovery research in recent years. The main focus of such analyses has been M1 and the corticospinal tract (CST), the main outflow tract of the human motor system.^6^ More recently, motor networks such as alternate corticofugal motor fibres, cortico-cerebellar networks and networks on the cortico-cortical level have gained an increasing interest in the field. For the latter and similarly to functional imaging data, most studies have addressed structural brain changes in frontal motor areas.

**Figure 1:**
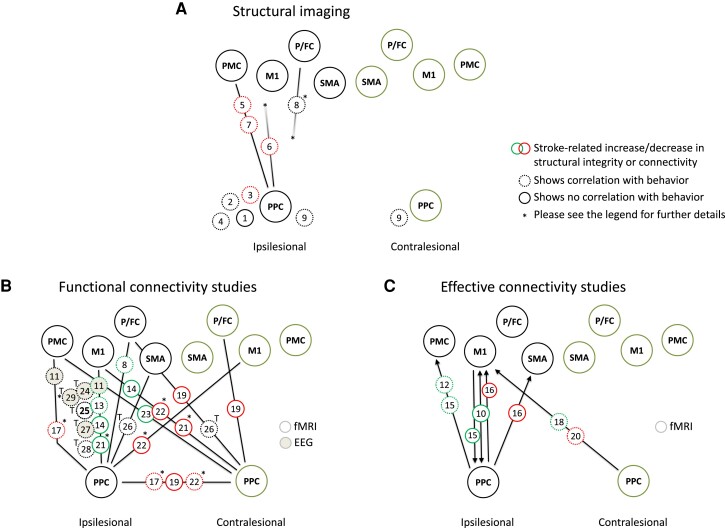
**Synopsis of structural imaging studies and functional and effective connectivity studies focusing on posterior parietal cortical areas and their contribution to motor recovery after stroke.** Green and red circles indicate increase or decrease in regional volumetric measures or parameters of tract/connection integrity (**A**), in parameters of functional (**B**) and effective (**C**) connectivity. For **B** and **C**, fMRI and EEG studies are indicated, respectively. Results are largely based on group comparisons with healthy controls, if not stated otherwise in selected cases (indicated by asterisks). Asterisks also indicate relevant methodological information. Dotted circles indicate a significant association with behaviour, e.g. residual motor functions, recovery over time or improvement during specific treatment paradigms (indicated by T). Please note that the direction of the association is not given by this synopsis. The ipsilesional and contralesional hemisphere is given. Selected studies (this legend's numbers in brackets refer to the numbers in circles in the figure, first author and year of publication are given): (1) Graterol Perez 2022,^42^ (2) Abela 2012,^44^ (3) Abela 2015,^43^ (4) D’Imperio 2021,^45^ (5) Schulz 2015,^22^ (6) Jacquemont 2022 ^46^ (no further details about seed/target regions of the superior longitudinal fascicles investigated), (7) Schulz 2017,^47^ (8) Hordacre 2021 ^48^ [reference: patients with large CST damage (versus. small) or better recovery (versus worse)], no precise tractography, (9) Koch 2021,^50^ (10) Schulz 2016,^51^ (11) Bönstrup 2018,^52^ (12) Hensel 2021,^33^ (13) Backhaus 2021,^53^ (14) Zhang 2016,^54^ (15) Liu 2022,^55^ (16) Inman 2012,^56^ (17) Hensel 2022 ^41^ (reference: patients with poor outcome versus good), (18) Pool 2018,^34^ (19) Allart 2020,^57^ (20) Zhao 2016,^58^ (21) Park 2011 ^59^ (selected time point for comparison: 6 months after stroke), (22) Lee 2018 ^60^ (selected time point for group comparison: 2 weeks after stroke, please note that decrease in FC was also detected for interhemispheric PPC-PMC and PPC-SMA connections, these alterations are not illustrated for the sake of clarity), (23) Wang 2010,^61^ (24) Wu 2015,^62^ (25) Wang 2020,^63^ (26) Lau 2021,^64^ (27) Cassidy 2021,^65^ (28) Kraeutner 2021,^66^ (29) Zhou 2018 ^67^ (correlation with training effect, but not with baseline motor performance). Please see the text for full citations. PPC, posterior parietal cortices; M1, primary motor cortex; PMC, premotor cortices; SMA, supplementary motor area; P/FC, prefrontal and frontal cortices (not further specified for illustration purposes).

More recently, studies have increasingly explored posterior parietal brain regions and their structural connections with frontal motor areas. For instance, it has been shown that subcortical strokes lead to atrophy and cortical thinning not only in ipsilesional M1, retrogradely affected by the lesion, but also in posterior parietal cortical areas along the IPS.^42^ Ipsilesional cortical grey matter atrophy in the angular gyrus and the SPL has been linked with unfavourable recovery in a longitudinal study.^43^ Patients exhibiting lesions which extended from the primary sensorimotor cortices into more parietal areas have been reported to be at risk of retaining more severe chronic motor deficits.^44^ Similar findings have been obtained by a recent lesion mapping study in chronic stroke patients which correlated lesions affecting white-matter tracts below posterior parietal cortical areas with reduced treatment gains in motor rehabilitation.^45^ Structural connectivity analyses have also been conducted to investigate to what extent the integrity of motor pathways linking posterior parietal cortical areas with key areas of the frontal lobe might influence recovery. For example, one study in 26 chronic stroke patients reconstructed parieto-frontal motor connections between the ipsilesional anterior IPS and the ventral PMC and found that the structural integrity of this connection positively correlated with recovered hand function in addition to the well-established influence of the integrity of the CST.^22^ Likewise, in a longitudinal setting, another study reconstructed the superior longitudinal fascicle and reported a positive correlation between its microstructural state after three weeks and recovery until twelve weeks.^46^ Other studies have argued that such structure–behaviour relationships might be particularly relevant in more severely impaired patients, e.g. with larger lesion loads to the CST.^47,48^ Such findings have been recently extended by analyses linking the connectivity of specific brain areas within larger brain networks. One analysis trained support vector machine classifiers to separate patients with natural recovery, as defined by the proportional recovery model,^49^ from patients without natural recovery. The authors found that a stronger connectivity between ipsilesional posterior parietal cortices with the global connectome, assessed two weeks after stroke, was positively associated with a larger likelihood of exhibiting subsequent natural recovery.^50^ A synopsis of structural analyses including structural connectivity studies is given in Fig. 1A.

### Functional and effective connectivity analyses of posterior parietal cortical areas

Aside from structural analyses, a variety of functional imaging studies have investigated the inter-regional interplay of posterior parietal cortical areas with other areas of the distributed motor network, particularly in the frontal lobe. Methodologically, functional connectivity (FC) analyses can be differentiated from effective connectivity (EC) analyses. FC describes the temporal relationship between spatially distant neurophysiological events. Most calculations are based on correlations between time series of functional MRI BOLD data or correlations of oscillations between different brain regions in task-free EEG data. Coupling strengths, e.g. between different motor areas, are non-directional and do not inform about the direction of interactions or causality. In contrast, EC calculations are mostly derived from task-related time series data and carry directional information about how brain activity in one region might influence the activity in another region.^68^

There are already some studies which have provided valuable insights into ipsilesional parieto-frontal connectivity after stroke. In well-recovered chronic stroke patients, task-related functional MRI combined with dynamic causal modelling for EC analysis found an increase in reciprocal information flow between ipsilesional anterior IPS and M1 during a simple hand grip task. However, the extent of this upregulation could not be linked to persistent motor deficits.^51^ A subsequent EEG study in a similar cohort of patients, three months after stroke, has found a significant upregulation in task-related FC between the same regions, the ipsilesional anterior IPS and M1. Moreover, patients with larger deficits exhibited stronger parieto-frontal communication.^52^ Further MRI studies provided additional insights particularly for the acute stage and patients with severe deficits.^33,48,53-55^ Well-recovered stroke patients, measured acutely after stroke, exhibited a significant EC from ipsilesional AIP to dorsal PMC modulated by hand tapping which was not evident in healthy controls. Moreover, the strength of this parieto-frontal coupling could predict the effect of a TMS perturbation paradigm of the contralesional anterior IPS which was found to improve hand motor performance, potentially via the modulation of interhemispheric coupling.^33^ Another MRI study investigated severely impaired acute stroke patients. These patients exhibited a strong upregulation in parieto-frontal FC between ipsilesional anterior IPS and M1. Importantly though, the extent of this early upregulation was associated with larger persistent deficits after three to six months.^53^ It has been speculated that posterior parietal cortical areas upregulate after stroke and activate their interactions with frontal key motor areas to support motor output. However, this upregulation seems to be largely insufficient since a persistent upregulation over time, particularly in severely impaired patients, has been evidenced by FC^54^ and recent EC analyses.^55^

However, drawing conclusions on the impact of the parietal cortices in motor recovery after stroke remains a challenge, as current studies give a complex picture of parieto-frontal interactions after stroke (see Fig. 1B-C for a synopsis). For example, in chronic stroke patients, some studies have reported a decrease in effective coupling from ipsilesional parietal cortices to M1 and SMA in severely impaired patients compared to healthy participants^56^ or have found a reduced FC between ipsilesional anterior IPS and dorsal PMC in less-recovered patients when compared to patients with a good outcome.^41^ Another study combined functional and structural imaging and TMS and observed that patients with an extensive lesion load to the CST exhibited higher FC in ipsilesional parieto-frontal networks.^48^ Patients with extensive CST damage showed a positive correlation between parieto-frontal FC and motor performance which might contrast with previous studies.^52^

In comparison to the ipsilesional hemisphere, contralesional or inter-hemispheric parieto-frontal connectivity data are still limited. One study in well-recovered patients found an increased information flow from contralesional SPL to ipsilesional M1 during hand movements.^34^ Other studies reported FC reductions within contralesional parieto-frontal networks^57^ or EC reductions from contralesional SPL onto ipsilesional M1 particularly in more severely affected stroke patients.^58^ One double-pulse TMS experiment has addressed the connectivity between the contralesional posterior parietal cortex and contralesional M1 and has not found any alterations after stroke nor any associations with motor function.^69^ Finally, few longitudinal studies, mostly conducted in small cohorts, have addressed temporal dynamics of parieto-frontal connectivity after stroke and have found rather variable increases and decreases in FC for parietal cortices, e.g. between ipsilesional motor and bilateral posterior parietal brain regions which did not show any connectivity–outcome relationships.^59-61^

Connectivity–behaviour relationships for ipsilesional parieto-frontal connectivity have been investigated for motor training after stroke. One EEG study has assessed changes in resting-state FC between frontal motor and multiple other brain regions during an intervention of home-based upper limb training over 28 days. It found that functional improvement was accompanied by decreases in FC between ipsilesional parietal cortices and M1 while FC values between M1 and PMC increased at the same time.^62^ On a speculative note, training might (re)-activate frontal premotor–motor networks which in turn could result in a reduced need for additional parietal input, i.e. a re-balancing of parieto-frontal and frontal functional motor networks could parallel motor training after stroke. Similarly, most likely supportive contributions have been previously suggested for prefrontal cortices by FC analyses for the contralesional hemisphere^59^ and by EC analyses during a motor imagery task for the ipsilesional hemisphere.^70^ Again and in line with brain activation studies, these considerations remain speculative as they remain associative in nature and do not prove any causality. Interventional studies probing the up- or downregulation of specific connections and analysing treatment effects would be needed to investigate whether parieto-frontal connectivity would exert driving influences or show specific or even unspecific changes because of training. Thereby and to make the situation even more difficult, parieto-frontal network involvement might be treatment specific. One study has investigated the addition of motor imagery training in chronic stroke patients and has found an upregulation of coupling strengths between ipsilesional parietal areas and M1 under motor imagery when compared to conventional rehabilitative treatment over four weeks.^63^ Another study has explored brain computer interface–guided robotic hand training and has found a treatment-associated increase in FC between bilateral SPL and ipsilesional SMA.^64^ Finally, the prediction of treatment gains has also been explored by several studies. For instance, one resting-state EEG study conducted early after stroke has reported that stronger baseline FC in the high beta band between ipsilesional posterior parietal leads and M1 positively contributed to motor improvement while stronger coupling between M1 and SMA was negatively related to functional improvement.^65^ A similar association between stronger baseline coherence in the high beta band between ipsilesional parietal leads and M1 has been related to improvement in a visuomotor tracking paradigm.^67^ Another study investigated a complex, gamified reaching task in the chronic stage of recovery. The training led to an increase in FC between left supramarginal gyrus and left SMA. The treatment effect itself was positively associated with the coupling strength between left angular gyrus and left M1.^66^ As a consequence, future studies will have to acknowledge that precise characteristics of experimental setups, motor task or training paradigms as well as outcome measures have a relevant influence on brain–behaviour associations, whether causal or not. An overview of functional and effective connectivity studies is given in Fig. 1B-C.

## Limitations

The aim of the present work was to provide a scoping review of posterior parietal cortical areas and their contribution to recovery after motor stroke. Three limitations are worth noting: First, the authors do neither claim any completeness nor have they utilized any objective assessment of the methodological quality of the selected studies. Hence, particularly the small sample sizes should be considered with caution when inferring any conclusions from the presented data. Second, study selection was biased towards the motor domain and, for connectivity studies, towards parieto-frontal connections. Studies with a focus on other domains of deficits, such as aphasia or neglect, and connectivity within posterior parietal brain regions themselves^38^ were not in the focus of the present work. For aphasia and neglect, the interested reader is referred to relevant and informative primary research and reviews.^71-77^ In this regard, the network–outcome inferences in the present report are dominated by motor aspects. However, neglect and aphasia are critical influential factors for recovered motor functions and the success of rehabilitation.^78^ Hence, to what extent the associations between posterior parietal cortical areas and recovery after motor stroke might be mediated by an impact on neglect and speech disturbances remains undiscussed. An integrative review covering all domains would be warranted to address this further and systematically. Third, the authors fully recognize that the posterior parietal cortex comprises multiple functionally and structurally defined areas. Also, findings were organized for the ipsilesional and contralesional hemisphere, but not for the right and left, or dominant and non-dominant hemispheres, an approach that might be particularly intuitive for language recovery research. Thus, the picture presented in this work, particularly the synopsis given in Fig. 1, is simplified for the sake of clarity and readability. The interested reader is advised to consult the primary literature for precise functional and anatomical information including hemisphere effects and dominance, coordinates of activation, tasks and paradigms, or seed/target areas of connectivity.

## Summary and future perspectives

Collectively, there is a growing body of literature indicating that posterior parietal cortices and their interactions to other motor areas clearly undergo stroke- and treatment-related changes that are associated with motor recovery processes. The present synopsis extends previous data on brain activity and connectivity changes after stroke which have largely focused on key motor areas of the frontal lobe.^4^ As discussed, the present picture appears rather complex and simple conclusions are still difficult to draw. It was not the aim of the present work to provide novel mechanistic or causal explanations of how precisely posterior parietal cortical areas may influence motor recovery after stroke. Brain–behaviour associations derived from functional and structural brain imaging remain associative in nature and do not evidence any causality. Notwithstanding these difficulties, the review aims at providing an integrative, illustrative and informative synopsis over the current body of literature to help other researchers to detect yet hidden patterns of alterations in the parieto-frontal network, to develop novel hypotheses, and to design appropriate studies which will have to combine, at best, imaging, behaviour and interventional approaches. For instance, the literature suggests that posterior parietal cortical areas might be most likely recruited in patients with severe deficits and higher lesion loads. On a speculative note, in these patients, the core motor networks appear to exhibit a high demand for additional neuronal input from prefrontal and posterior parietal areas, potentially to enhance excitability and promote plasticity to compensate for the motor network disruption. However, whether changes in posterior parietal brain activity and connectivity are causally related to recovered function or even linked to long-term recovery, i.e. whether they might drive plasticity and recovery, remains unclear. If not, an unspecific epiphenomenon of the *upregulated brain* must be considered as well. There are different ways how future studies could investigate these alternative hypotheses. For instance, short interventional studies could use TMS or transcranial direct current stimulation (tDCS) to perturbate or up- and downregulate ongoing activity in posterior parietal brain areas and analyse subsequent changes in primary motor cortical excitability. Herein, the combined analysis of patients with minor and severe deficits could then inform about different causal roles of these areas in specific patient cohorts. When conducted at different time points, early or late after stroke, the aspect of time dependency could be addressed as well. Finally, when it comes to brain–behaviour relationships, studies will have to acknowledge that the selection of motor tasks, from simple tasks to very complex tasking including reach and grasp components, will have an important impact on activation, thereby influencing the susceptibility to perturbation. As another point, data in severely impaired patients with unfavourable outcome have indicated that a persisting overactivation in posterior parietal cortical activity or coupling over time might reflect a supportive, but futile attempt of the brain to activate available resources. However, there are also some TMS data which suggest that posterior parietal areas might be engaged in maladaptive processes. To better understand these discrepancies of supportive or maladaptive roles, we need prospective, interventional and well-powered TMS/tDCS studies on samples covering a broad spectrum of impairment from minor deficits to more severe impairment. Ultimately, to what extent an early additional upregulation of posterior parietal cortical areas, e.g. by means of activating TMS or tDCS,^79^ might promote future recovery in clinical cohorts has to be investigated prospectively and systematically. So far, appropriate cohorts have not been studied yet and posterior parietal stimulation data in clinical cohorts are limited.^33,40,41,80,81^
